# Using a household-structured branching process to analyse contact tracing in the SARS-CoV-2 pandemic

**DOI:** 10.1098/rstb.2020.0267

**Published:** 2021-07-19

**Authors:** Martyn Fyles, Elizabeth Fearon, Christopher Overton, University of Manchester COVID-19 Modelling Group, Tom Wingfield, Graham F. Medley, Ian Hall, Lorenzo Pellis, Thomas House

**Affiliations:** ^1^ Department of Mathematics, University of Manchester, Manchester M13 9PY, UK; ^2^ The Alan Turing Institute, London NW1 2DB, UK; ^3^ Centre for the Mathematical Modelling of Infectious Disease, London School of Hygiene and Tropical Medicine, London WC1E 7HT, UK; ^4^ Department of Global Health and Development, London School of Hygiene and Tropical Medicine, London WC1E 7HT, UK; ^5^ Department of Clinical Sciences and International Public Health, Liverpool School of Tropical Medicine, Liverpool L3 5QA, UK; ^6^ Tropical and Infectious Disease Unit, Liverpool University Hospitals NHS Foundation Trust, Liverpool L7 8XP, UK; ^7^ WHO Collaborating Centre on Tuberculosis and Social Medicine, Department of Global Public Health, Karolinska Institutet, 171 77 Stockholm, Sweden; ^8^ Public Health England, UK; ^9^ Joint UNIversities Pandemic and Epidemiological Research, https://maths.org/juniper/, Daresbury WA4 4AD, UK; ^10^ IBM Research, Hartree Centre, Daresbury WA4 4AD, UK

**Keywords:** contact tracing, SARS-CoV-2, COVID-19, infectious disease, epidemiology, epidemic

## Abstract

We explore strategies of contact tracing, case isolation and quarantine of exposed contacts to control the SARS-CoV-2 epidemic using a branching process model with household structure. This structure reflects higher transmission risks among household members than among non-household members. We explore strategic implementation choices that make use of household structure, and investigate strategies including two-step tracing, backwards tracing, smartphone tracing and tracing upon symptom report rather than test results. The primary model outcome is the effect of contact tracing, in combination with different levels of physical distancing, on the growth rate of the epidemic. Furthermore, we investigate epidemic extinction times to indicate the time period over which interventions must be sustained. We consider effects of non-uptake of isolation/quarantine, non-adherence, and declining recall of contacts over time. Our results find that, compared to self-isolation of cases without contact tracing, a contact tracing strategy designed to take advantage of household structure allows for some relaxation of physical distancing measures but cannot completely control the epidemic absent of other measures. Even assuming no imported cases and sustainment of moderate physical distancing, testing and tracing efforts, the time to bring the epidemic to extinction could be in the order of months to years.

This article is part of the theme issue ‘Modelling that shaped the early COVID-19 pandemic response in the UK’.

## 1. Background

The COVID-19 pandemic, arising from infection with SARS-CoV-2, has rapidly spread across the world leading to significant loss of life, with many different interventions employed in attempts to reduce the spread of the disease. To avoid overwhelming the capacity of healthcare systems and to limit mortality, many countries, including the UK, adopted policies to dramatically reduce the number of contacts via which infections could occur [1]. These ‘lockdown’ policies have included: requiring all but essential workers to stay physically in their residences; schools, universities, entertainment venues and all but essential businesses to close; and limitations on outside-household activities and meetings. In many countries, including the UK, there were dramatic curtailments in the number of outside-household social contacts made across most of the population, but household contacts remained [1]. While effective in reducing epidemic growth, strict lockdown policies are not sustainable economically and socially [2]. As such regulations are relaxed, workplaces, schools and businesses reopen (albeit with additional safety measures), with increased contact between households occurring. In this context, the role of contact tracing and isolation is to target quarantine to households with higher risk of infection to allow increased economic and social interaction without a return to rapid epidemic growth.

Contact tracing policies consist of three main components: *isolation* of identified infected individuals to prevent onwards transmission, *tracing* of their recent contacts who might have been exposed to infection, and the intervention, most commonly quarantine, applied to the traced individuals to try to halt the chains of transmission. As of August 2020 in the UK, a contact is defined as a case's household member or a sexual partner and/or someone with whom they have: had skin-to-skin contact; coughed on; been within 1 m of for more than 1 min; had a face-to-face conversation with within 1 m; been within 2 m of for at least 15 min; or shared a vehicle with (or sat near if a plane or large vehicle) [3]. In the UK, case isolation and contact quarantine is done at home, even though individuals are advised to distance themselves from household members. Given the likely difficulties in achieving this in practice, case isolation and contact quarantine policies will likely not prevent within-household transmission once an infection is introduced, but rather are aimed at reducing transmission between households.

Previous research on other respiratory infections finds households to be key structures in understanding population level transmission dynamics [4]. The nature and extent of contact between household members increases the likelihood of transmission compared to other types of social contacts. Households bring together disparate social networks such as those of different workplaces and schools, and they often bring together people of different age groups via family relationships [5]. For SARS-CoV-2, emerging evidence is that household contacts of cases are much more likely to become infected than non-household contacts and a systematic review estimated household secondary attack rates to lie between 15.4 and 22.2% [6]. However, the reliance on symptomatic diagnosis of cases in many studies means this is likely to be an underestimate. Household structure should also benefit the contact tracing process in that household contacts are much more easily identified, so models or policies that do not account explicitly for this structure might be less accurate in their conclusions or less efficient, respectively, than those that do. For example, given that negative serial intervals have been observed for SARS-CoV-2 [7], it is possible that a non-index case in a household epidemic develops symptoms before the index case, leading to a reduced time until symptoms are reported for the household-structured branching process, compared to an individual-level branching process where symptoms must be reported at each step.

Here, we use a household-structured model to explore the potential impact of contact tracing, isolation and quarantine on epidemic growth, considering a range of social contact, transmission, tracing performance and population adherence assumptions. We model the effects of potential strategies to improve contact tracing effectiveness and consider the timeframe over which interventions must be kept in place to achieve infection extinction in a closed population assuming no importations, which may be difficult to achieve in practice.

## 2. Challenges to contact tracing and isolation

There are a number of challenges to using contact tracing and isolation to suppress SARS-CoV-2 transmission. Early models, even those made prior to upwards revision of unconstrained growth estimates [8], suggested that contact tracing must operate with minimal delay and with high levels of accuracy (70–90% of contacts traced for R0 2.5–3.5) [9,10] if it is to interrupt enough transmission chains to achieve control. The first step of identifying an index case is challenging for SARS-CoV-2; many cases are asymptomatic, and symptomatic cases frequently have mild or non-distinguishing initial symptoms. Once identified, the index case is isolated and subsequently interviewed to identify all individuals who may have been exposed while the index case was infectious, which becomes more difficult the longer the pre-symptomatic infectious period. Asymptomatic cases will not become index cases unless some testing is performed regardless of the individuals' symptom status. Some subpopulations, such as healthcare workers, might receive regular testing regardless of their symptom status, and asymptomatic cases may become index cases through this avenue, but this is the exception rather than the rule. The contact tracing interview with an identified case is subject to the delay between infection and symptom onset and/or case confirmation via testing, plus any further procedural delay to the interview. The task of reaching the contacts is also subject to delays and potentially impossible when they are non-identifiable (e.g. strangers to the case), unreported (e.g. if the case fears disclosure of their contacts or the contact tracing process is not adapted to the needs of the population [11]) or unreachable. For a new infection, it is also possible that the contact definition used for tracing is not well matched to the main modes of transmission. Traced contacts are then asked to quarantine to restrict the potential spread of the infection and are potentially monitored for signs of infection. If a quarantined contact meets the case definition or develops symptoms, their contacts prior to quarantine are in turn then the subjects of tracing attempts.

Timing of symptom onset versus infectiousness is important [12,13]. Control of SARS-CoV-1 was facilitated by the fact that peak infectiousness occurred after the onset of noticeable symptoms and that there was little pre-symptomatic or asymptomatic transmission [14,15]. For SARS-CoV-2, significant pre-symptomatic transmission means that by the time an infected individual is identified, there is a high probability that they have already infected others [16–18]. Many infected people are asymptomatic or experience mild symptoms, remaining undiscovered [19]. This issue is compounded by limited testing and high rates of false negative in tests to diagnose active infections [20].

In the UK, as of September 2020, contacts of identified cases are asked to quarantine for a period of 14 days following contact with the case (within which the vast majority of incubation periods would occur [21]). If the contact develops symptoms and tests positive, they must self-isolate for 10 days and their contacts, including those in their households, must quarantine for 14 days from the time of their symptom onset [22]. Particularly in the absence of contact testing and release from quarantine if negative, there will be a proportion of society that is unable or unwilling to take up or adhere to issued notices to quarantine or to isolate for the full period. The effects of non-uptake or of partial adherence on epidemic growth will depend on the dynamics of within-household transmission, making this structure important to capture explicitly in modelling. There might also be practical trade-offs since a strict system that in theory might be most effective in reducing transmission could result in lower adherence; and there are complications to releasing contacts early after a negative test when the test has low sensitivity [23]. While there is evidence that the average number of contacts made at the beginning of the UK's first ‘lockdown’ at the end of March 2020 was reduced by over 70% [1] compared to a 2006 contact survey [15], another survey from 2 days in early May 2020 found that almost three quarters of individuals who showed or whose household showed SARS-CoV-2 symptoms had left the house in the previous 24 h, behaviour that was contrary to policy at the time [24]. Across past studies, reporting of adherence to quarantine orders varies widely, and generally involves shorter time frames than those involved in the current pandemic. Further, we would expect adherence to vary over time as the perceived risk of breaking quarantine, public trust in health services and government, and the benefits of breaking quarantine (such as socializing, which may be impossible under lockdown) change over time. In practical terms, this means that we will not know what adherence levels might be expected over the time period necessary for control of SARS-CoV-2 in the absence of vaccination [25].

## 3. Contact tracing, isolation strategies and considerations post-lockdown

Those countries that have maintained periods of SARS-CoV-2 epidemic suppression have used contact tracing, isolation and quarantine measures alongside a variety of other interventions [26,27], and have re-deployed stricter physical distancing again in the event of rising cases. This experience is consistent with modelling that suggests that such suppression requires contact tracing to be implemented alongside policies that reduce usual patterns of social contact [28].

There are modifications to the contact tracing process that could improve efficiency and effectiveness. The strong likelihood of pre-symptomatic transmission within the household means that tracing not only the index case's contacts but contacts of the whole household, and quarantining the whole household of a traced contact might be effective. Immediate quarantine of contacts rather than monitoring for symptom development contributes to prevention of asymptomatic transmission and pre-symptomatic transmission if tracing occurs quickly enough [29]. Immediate tracing and quarantining of contacts-of-contacts—‘two-step tracing’, implemented for some contacts as part of Vietnam's strategy [26]—could further improve the speed of the control process relative to transmission, but at the cost of a large number of people being quarantined per case, which if widely applied may lead to effectively placing small areas in lockdown [30]. In the long run, this initial cost might be worthwhile if better epidemic control is achieved. Delays due to testing can be eliminated if tracing is initiated upon symptom reporting, though in practice many with symptoms will not actually have SARS-CoV-2 and a sizable proportion with SARS-CoV-2 will have no symptoms [31]. To reduce tracing delays and increase the number of contacts who can be identified and traced, the use of smartphone apps that record proximate devices (a proxy for contacts) and can send them notifications to quarantine upon a case testing positive, have been explored in a number of settings and trialled on the Isle of Wight [32]. Modelling suggests that such an app could potentially bring the effective reproduction number below one, but only if population uptake is very high [10]. In the UK, this would equate to at least 80% uptake by smartphone owners [10], which is higher than that yet achieved elsewhere. Fears over privacy, stigma and misinformation could impede uptake [33]. In countries which have deployed contact tracing apps the uptake is very mixed; in early September 2020, data on app uptake was collected from news articles for 30 countries, with 19/30 countries achieving uptake of less than 10% [34].

**Figure 1.**
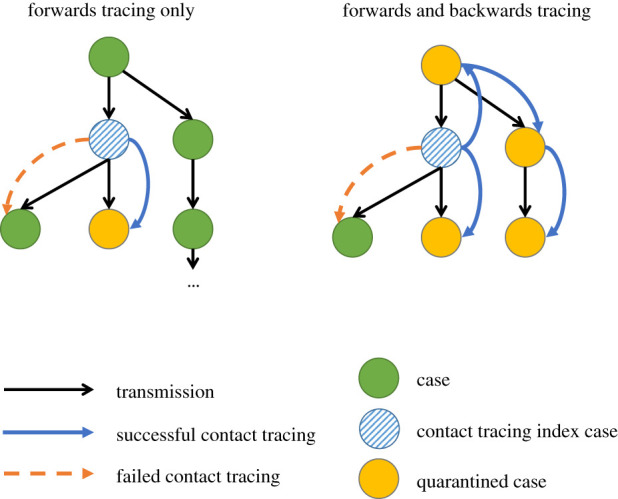
An illustration of backwards and forwards tracing. Here, the chain of transmission is represented using black arrows. The blue circle represents the first case in this chain to be detected. The left-hand contact tracing chain contains only forwards contact tracing and as a result only the infectees of the index case are traced. The right-hand plot has the same forwards tracing as before, and a backwards contact tracing event in which the infector of the index case was discovered, and forwards contact tracing then enables sibling infections to the index case to be traced and quarantined. (Online version in colour.)

In contact tracing, there is an implicit form of direction—an individual is said to be forward contact traced if the direction of contact tracing is the same as the direction of transmission, which results in the traced individual being an infectee of the case from whom the tracing attempt was initiated. Backwards contact tracing is when the direction of tracing is opposite to the direction of transmission, which results in the traced individual being the infector of the case who initiated the tracing attempt. For some diseases, backwards tracing can be an important strategy as a backwards step can then be followed by a forwards tracing step again to discover ‘sibling’ infections who share the same infector [35]. A visualization of backwards and forwards tracing is plotted in figure 1, where a successful backwards tracing event leads to the quarantine of a sibling chain of transmission.

One implementation of backwards tracing involves increasing the time window prior to symptom onset over which contacts are traced to include the likely date of the case's infection, in addition to covering their likely infectious period. Backwards tracing might be especially efficacious due to a biased sampling effect: the more secondary infections an individual causes, the more likely they are to be isolated through backwards tracing initiated by one of their secondary infections. Therefore, backwards tracing has an increased likelihood of finding individuals who caused a lot of secondary infections, and for these individuals it might then be highly worthwhile *forwards* tracing to find their infectees [36]. Following this logic, if the number of secondary cases is highly overdispersed, as has been estimated [37], then backwards tracing could be especially useful to help identify ‘superspreading’ events involving large numbers of transmissions [35]. However, tracing backwards to move forwards again might be less useful when tracing delays are high relative to the speed of transmission [38], and when accurate recall or identification of contacts degrades over longer time frames.

If contact tracing is adopted as a strategy to help achieve local elimination, then to inform decision-making, we need to understand the time scales over which contact tracing and concurrent physical distancing interventions must be sustained to reach elimination (in the absence of new importations from outside), without which transmission is likely to climb again. Even if interventions are enough to drive the infection to elimination, we might expect a long ‘tail’ of ongoing but increasingly sporadic transmission during which time reintroduction of the epidemic is an ongoing risk if tracing and physical distancing measures are relaxed [39].

Here we consider what role contact tracing, isolation and quarantine might play in suppressing epidemic growth across a range of plausible epidemiological and behavioural assumptions. We begin more generally and then consider the case of the UK more specifically during May – June 2020, considering lockdown easing scenarios and the contact tracing, isolation and quarantine policy adopted on 28 May 2020. To capture the interplay of within- and between-household epidemic dynamics [40], we use a household-structured branching process model to investigate how contact tracing and isolating might affect the growth rate, the probability of epidemic extinction and extinction times for: contact tracing strategies performed at the household or individual level; different levels of physical distancing; assumptions about the probability of discovering infected individuals; a range of suggested improvements to contact tracing, including an app to reduce tracing times and improve the probability of identifying and reaching contacts. We also examine how key aspects of implementing the interventions, as well as uptake and adherence to them among the population, might affect their efficacy in practice.

## 4. Methods

### (a) Model

#### (i) Household-structured branching processes and contact tracing

We model the SARS-CoV-2 epidemic as a branching process of infections of individuals grouped into households [41]. Transmission is considered on two levels: *local* within-household transmission and *global* between-household transmission. We assume a static population distribution of household sizes, excluding migration in and out of households, and that infection can be introduced into a household only once. In mathematical terms, a household-structured branching process is considered to be a multitype branching process, where the type of a case is the generation of the household epidemic they belong to, in combination with their household size. The number of offspring produced by a case is conditional upon the size of the household they belong to, and how many susceptibles remain in that household. Upon introduction of the infection to a household, a homogeneously mixing within-household epidemic is initiated, referred to as the *local epidemic*. Individuals infected by a local epidemic are able to propagate the infection globally by introducing the infection to a fully susceptible household. Individuals in the model differ only by the size of the household they belong to, and we do not model explicit household compositions (e.g. by age, gender).

The household-structured branching process is an approximation to an epidemic that is derived when the epidemic is considered to be spreading through a population of individuals segmented into households, with homogenous mixing between households, and homogenous mixing within households. If it is early in the epidemic, and there is little to no susceptible depletion then the homogenous mixing assumption implies that the probability the infection is reintroduced into a previously infected household is effectively zero, hence our assumption that the infection is only introduced into the household once. This effectively linearizes the transmission model at the level of households, and the model is therefore limited in that the approximation does not hold when there is significant global depletion of susceptibles, and for this paper we therefore take the population size to be infinite. As a result, the model always represents a scenario with no immunity. In the UK in May–June 2020, seroprevalence was estimated to range from 0.5% to 17.5% in the worst affected region, with immunity low overall in the UK [42].

As a result of the infection being introduced only once to each household, a tree-like structure of cases is created, which grows as the epidemic progresses, with links representing infections between individuals. From this tree-like structure of cases, we can also construct a distinct tree-like structure of infected households, where each link connects two households if and only if a global infection from a member of one household has infected a member of, and therefore introduced the infection in, the other household.

**Figure 2.**
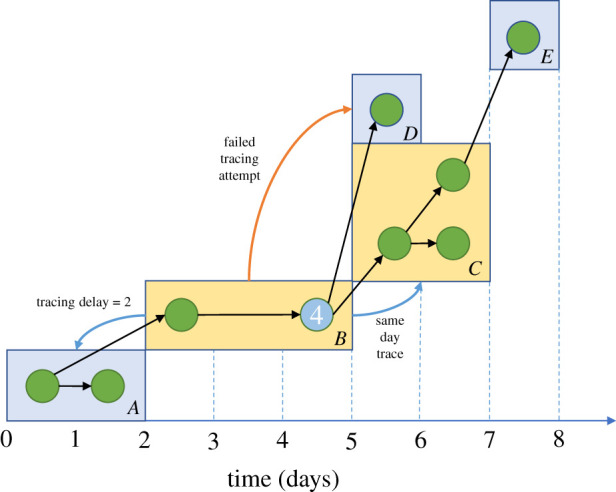
An illustration of the household branching process with contact tracing. Households are identified by letters in the bottom right-hand corner of each rectangle. The infection is discovered in case 4. This quarantines household B and initiates contact tracing of connected households A, C and D. The backwards tracing attempt to household A succeeds, with a time delay of 2 days. Household C is traced immediately, quarantining several cases early in their infection. When there is symptom onset in one of these cases the contact tracing process will propagate again by attempting to reach household E, potentially after a testing delay. The tracing attempt to household D did not succeed, and this household will continue to behave as normal and spread the infection until an intervention is applied through a different route. The *x*-axis refers to the temporal evolution of the transmission process in this example. (Online version in colour.)

This transmission tree generated by the branching process allows for modelling of contact tracing as a type of ‘superinfection’ along the tree; for a household-structured branching process the contact tracing process could be considered to superinfect at the level of households (figure 2). When the contact tracing process ‘superinfects’ a household or individual, then the effects of the contact tracing process are applied. These are typically: quarantine, resulting in a reduced ability of cases in the household to transmit the infection globally; and surveillance, where all individuals in the household are on the lookout for symptom onset.

The household structure introduces additional effects to the contact tracing dynamics that enable the exploration of contact tracing and isolation policies at the level of either individuals or households. Each infection in a household has an independent and identically distributed probability of being self-identified, and therefore, infection is more likely to be identified in larger households. Larger households tend to make more global contacts than smaller ones, and therefore may be responsible for spreading more of the infection but are also more likely to have an infection identified in them. Later generations of a local epidemic may be quarantined early in their infection due to a household member belonging to an earlier generation of the local epidemic self-identifying and reporting the infection.

At time 0, the infection is seeded by creating a number of initial infections with infectious age 0, with each infection being the sole index case in a household epidemic. In simulations where we estimate the epidemic growth rate, the first 10 days are discarded as model burn-in (see electronic supplementary material, figure S1), as we must allow the local within-household epidemics to progress, introduce the infection into new households and for the contact tracing process to be initiated by a symptomatic case self-identifying their infection. The epidemic growth rate is then estimated using log-linear regression on days 10–25 of the simulation.

#### (ii) Social contacts and infection

Each day, individuals make contacts, the number of which is sampled from an overdispersed negative binomial distribution (overdispersion parameter 0.32; see electronic supplementary material for technical details) estimated with data on ‘all contacts’ (i.e. physical and non-physical) from the 2006 POLYMOD study [5], stratified according to the size of the household in which the individual resides. Household sizes are aligned with the 2019 Office for National Statistics (ONS) survey [43], and are categorized as of size 1, 2, 3, 4, 5, 6+. Contacts are only distinguished by whether they are within (local) or outside (global) the household, with the proportion of each again estimated using POLYMOD. Given it is challenging to distinguish one long contact from separate short contacts between household members when measuring them, we assume at most one local contact per day with each other household member, which occurs each day with fixed probability that depends upon household size. As such, the number of local contacts is binomially distributed. The number of global contacts is given by the difference between the number of contacts made in total and the number of local contacts. If an illegal combination of contacts is observed (e.g. three local and two total), then all contacts are redrawn from their distributions until a legal combination occurs. We do not consider repeated global contacts, so individuals experience no susceptible depletion among those whom they might infect globally. We do not consider the extent to which each household member's global contacts overlap. That is, every global contact reaches a new individual that belongs to a new household. Again, this represents a worst case scenario for epidemic spread, although not necessarily for the efficacy of contact tracing [44].

Physical distancing and lockdown measures are modelled through a Bernoulli thinning of an individual's global contacts, with their local contact remaining unchanged, leading to a percentage reduction in global contacts made. For example, suppose that physical distancing is leading to a 50% reduction in global contacts, then we would draw the number of global contacts that an individual would make in the absence of physical distancing, and then consider each contact to occur with probability *p* = 0.5. This leads to a binomial number of actual contacts made conditional upon the number of trials being drawn from an overdispersed negative binomial (see §3.2 in the electronic supplementary materials for details). We explore physical distancing by applying the same reduction in global contacts to every individual, which we refer to as uniform physical distancing. Because we expect the level of physical distancing to have some dependence upon household size and composition in practice, we later assume lockdown relaxation scenarios and derive physical distancing parameters for these scenarios where the physical distancing reduction is conditional upon the size of the household.

Once the number of contacts of an infective has been drawn on each day, we compute the probability that such a contact is able to transmit the infection. Because the probability of a contact causing infection cannot be directly observed, we condition the probability that a contact spreads the infection upon the infectious age of the individual, in such a way that generation times are Weibull-distributed with a mean of 5 days and standard deviation of 1.92 days [10], where a generation time is defined as the delay from becoming infected to a transmission of the virus (see §4 in the electronic supplementary materials). The distribution of generation times is an important quantity to calibrate, as it defines a variety of timings and quantities within the model, for example, the infectious age and number of secondary infections generated by the time an individual tests positive. The probability of a contact leading to infection is tuned for the local and global settings such that the model has a baseline growth rate of *r* = 0.22 d^−1^ [8] and a household secondary attack rate of 21.4%, based on the mean of several studies that were available during early April 2020 [45–47]. The baseline scenario assumes that individuals activate self-isolation and household quarantine when they believe they are infected, but there is no active contact tracing process. Thus, any observed reduction in the growth rate can be attributed solely to the contact tracing process, and not to effects of the case's household quarantine.

#### (iii) Case identification and contact tracing delays

For contact tracing to be initiated, an infected individual first needs to be identified. If an individual develops symptoms, their incubation period, i.e. the time between their infection and onset of symptoms, is gamma distributed with mean of 4.84 days and standard deviation of 2.79 [21]. Given estimates for the probability of being asymptomatic ranges between 30 and 70% [31,48–50], we consider an upper limit in the untraced case self-identification probability of 50%, to account for mild cases that do experience symptoms but do not report them.

For untraced individuals, we assume a gamma distributed delay with a mean of 2.62 days and standard deviation 2.38 days between symptom onset and case self-identification, to account for symptom reporting delays. However, for contact-traced individuals we remove this delay, because traced individuals are aware they have been exposed and therefore are assumed to report infection at symptom onset without the symptom reporting delay. Case identification in a household that is not already quarantined leads to an immediate quarantine of all other household members, and we explore initiating tracing both immediately upon symptom report and waiting for a positive test result before initiating tracing. If tracing begins after a positive test, we add a further testing delay to account for the delay from swabbing to receiving results, which is gamma distributed with a mean of 1.54 days and standard deviation of 1.1, parametrized using a truncation corrected maximum likelihood estimator applied to anonymized UK line-list data [21,51].

Contacts are traced with a probability of success, and successfully traced individuals are assigned a tracing delay time from a Poisson distribution, with a mean value that we vary between 1.5 and 2.5 days. We explore a range of possible contact tracing success probabilities from 70 to 95%, the upper bound of which was the percentage of known contacts (i.e. those who could be identified) who were successfully traced by Public Health England during the containment phase [52]. At the end of the tracing delay, the successfully traced household is quarantined. If an individual is adhering to quarantine or isolation, we assume they make no global contacts at all, but within-household contacts continue. From CoMix [1], it appears that the frequency of household contacts does not increase when outside-household contacts reduce. Therefore, although we recognize the intensity of the contacts might change, in the absence of evidence we assume no increase in local transmission while a household is quarantined.

#### (iv) Contact tracing strategies

Our default contact tracing strategy is ‘household level’ and designed to take advantage of the household structure; when an infection is identified in a household all members of the household have their contacts traced. When a contact is successfully traced, their whole household quarantines. We compare this to an ‘individual-level’ contact tracing process in which only individual cases have their contact traced, and only the specific people identified as contacts and successfully traced are quarantined. The rationale behind household-level contact tracing is that once a case has been detected in a household, there is an increased likelihood that the other household members are already infected and have possibly spread the infection outside the household but have not had symptom onset or received a test result. Thus, household-level contact tracing would initiate contact tracing earlier in the infection for cases who were infected by a household member. The individual-level contact tracing model, with household structure affecting infection dynamics but quarantining of contacts implemented on an individual level, is closest to what was adopted in the UK in May 2020.

**Figure 3.**
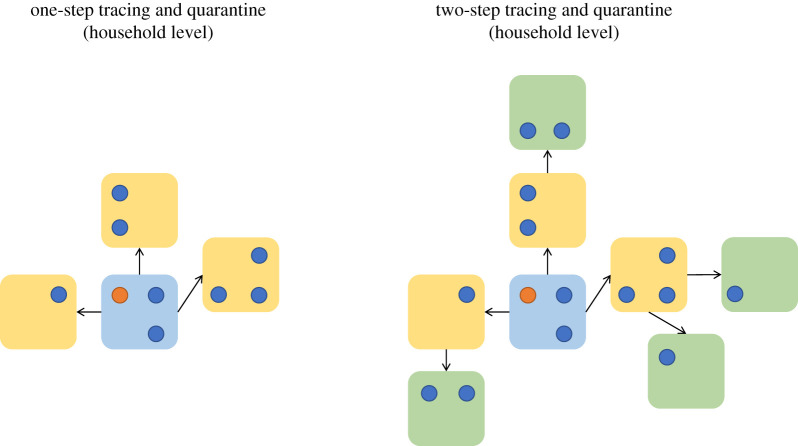
An illustration of the two-step tracing at the household level. We assume that all within-household contacts are always traced. For both one- and two-step tracing at the household level, all household members undergo quarantine and tracing regardless of whether they are the index case or not. In both of these examples, all of the individuals have been placed under quarantine or isolation once detected (for the index case) or traced. Households who were not successfully traced are not shown here. (Online version in colour.)

Given the speed of transmission, we further consider a ‘two-step’ contact tracing process at the level of households, as illustrated in figure 3. One-step contact tracing attempts to isolate individuals who are distance 1 from a known case, and two-step contact tracing attempts to isolate individuals who are distance 2 from a known case. As previously discussed, contact tracing can either occur at the household level or the individual level, and this leads to slightly different distances as the fundamental epidemiological unit is different. For household-level contact tracing, the distance between two individuals is defined in terms of the distances between their households: individuals who live in the same household are distance 0, individuals are distance 1 if they live in different households, but there has been contact between the households by any two members. Variations on this approach have been used in Vietnam for SARS-CoV-2 [26], although typically only on the level of individuals.

To consider a smartphone tracing app scenario [53], we allocate a random proportion of the population to be running the app on their smartphones, considering a wide range (table 2). When both individuals on the ends of a transmission event have been allocated the app, we assume any contact tracing attempts between these individuals succeed with 100% probability, and the tracing delay is reduced to zero. As both ends of a transmission event are required to have the app, the probability of seeing the app trace the transmission event is quadratic in the probability of an individual having the app. Here, we are considering a ‘perfect world’ contact tracing app and making the assumption that the app is able to record all epidemiologically relevant contacts. We do not allow for the possibility that the app misses a contact which causes a transmission.

In our models exploring the effect of adherence to quarantine, we consider (i) non-uptake of quarantine among traced contacts, implemented at the level of households; and (ii) declining adherence to quarantine over time, whereby a proportion of households have a propensity for declining adherence [24]. Among these households, each individual has a fixed probability of early exit from quarantine each day, at which point the individual then resumes making global contacts again.

A practical implementation of backwards tracing is considered, as it relates to the strategy implemented in the UK. The probability of including the infecting individual on a list of contacts to be traced depends on how many days pre-symptom onset is included as days when eligible contacts were made. For instance, the UK policy traces back 2 days in recognition of the significant role that pre-symptomatic transmission plays, but this is unlikely to include the infecting individual, given the distribution of incubation periods (with an approx. 5 day mean). To include the infecting individual as a potentially traceable contact, this time window would need to be up to 14 days. However, the further ago a contact was, the more difficult it might be to recall. A simple model of recall is applied, where the ability to recall a contact decays at a geometric rate as a function of the number of days since the contact occurred. There is little evidence to inform this parameter; our assumed parameters come from the personal experience of contact tracers. If the contact tracing is performed digitally through the app, then there is no recall decay and we explore the interaction between digital contact tracing and recall decay, as the increased speed of digital contact tracing also should allow for the sibling chains of transmission to be traced more rapidly.

### (b) Parameters

Parameter values used for our simulation and their sources are summarized in tables 1 and 2. We distinguish between several types of parameters: data-driven, uncertain and scenario parameters. Data-driven parameters (table 1), such as the household size distribution, are not varied in the sensitivity analysis. Other parameters, such as the probability of contact tracing success, are subject to significant uncertainty and we sample these from a prior distribution between simulation runs to perform a sensitivity analysis, as detailed in table 2. In several sections of this paper, we choose parameters that represent fixed scenarios and compare structural model variations without further parameter variation, to make the results more interpretable; for example, when we examine increasing the amount of backwards tracing that is performed then we keep the epidemic and contact tracing parameters fixed. The fixed parameters assumed when we are investigating backwards tracing are detailed in table 3. Our assumed scenarios of lockdown relaxations are detailed in table 4, and the resulting reduction in social contacts stratified by household size are described in table 5.

**Table 1.** Data-driven parameters.

parameter	values	source
baseline epidemic growth rate (pre-interventions)	0.22 per day (doubling time approx. 3 days)	[8]
incubation period	gamma (mean = 4.84 days, s.d. = 2.79 days)	[21]
generation time	Weibull (mean = 5.0, s.d. = 1.92 days)	[10]
household size distribution	1: 0.29, 2: 0.35, 3: 0.15, 4: 0.14, 5: 0.05, 6+: 0.02	[43]
onset to identification delay	gamma (mean = 2.62 days, s.d. = 2.38 days)	assumed using data from Singapore onset to visit to medical provider [8]
recall decay rate	10%	assumed from experience of contact tracers

**Table 2.** Sensitivity analysis parameters. PHE, Public Health England.

parameter	value	source	sensitivity analysis distribution
reduction in global contacts per day due to physical distancing	0–90%	lockdown reduction around 90% [1]	uniform (0.0, 0.9)
probability of untraced case self-identification	0.1, 0.2, 0.3, 0.4, 0.5	bounded using asymptomatic infection probabilities	equal probability
testing delay (from identification, isolation and specimen collection to test result)	gamma distributed, with mean varied between 1.5 and 2.5 days, having a fixed standard deviation of 1.11 days. (only applies to simulations that require testing before tracing)	estimates from PHE anonymized line-list data	mean varied between 1.5 days and 2.5 days
tracing delay (from identification and isolation of infector to effect of tracing on infectee)	Poisson with mean distributed between 1.5 and 2.5 days	estimates from PHE anonymized line-list data	Poisson parameter∼uniform (1.5, 2.5)
probability of contact tracing success for global contacts (in the absence of an app)	70–95% based on 95.2% of all *identified* contacts successfully traced during UK containment period	PHE containment period contact tracing [52]	uniform (70, 95)
proportion of population with a smartphone and with app installed	0–50%	Singapore's Trace Together uptake of approximately 40% (Sept 2020) [54]. Other apps reporting lower uptake [34].	uniform (0, 0.5)
uptake of quarantine among traced households	50–100% (only applies to simulations where non-adherence is allowed)	assumed	uniform (0.5,1)
proportion of households that have the propensity to not adhere to full quarantine stay	0–50% (only applies to simulations where non-adherence is allowed)	assumed	uniform (0, 0.5)
daily probability to leave isolation or quarantine early (if household has the propensity to not adhere to full quarantine stay)	0–5% (only applies to simulations where non-adherence is allowed)	assumed	uniform (0, 0.05)

**Table 3.** Model parameters for the simulations where we only vary the number of days prior to symptom onset are traced.

parameter	value
untraced case self-identification probability	30%
contact tracing success probability	80%
mean contact tracing delay	2 days
reduction in global contacts	30%
mean testing delay	1.5 days

**Table 4.** Assumed lockdown relaxation scenarios. The baseline number of cases is calibrated to the England lockdown of March–April 2020 [1], and we consider increasing the number of contacts that occurred.

scenario	effect on different contact types		
	workplace contacts	school contacts	leisure contacts
A	20% increase	10% resume	0% resume
B	30% increase	25% resume	10% resume
C	30% increase	50% resume	10% resume
D	40% increase	60% resume	30% resume
E	50% increase	100% resume	75% resume

**Table 5.** The global contact reduction relative to POLYMOD for the scenarios described in table 4. For these scenarios, we apply a reduction in global contacts that is stratified by household size.

household size	global contact reduction relative to POLYMOD (%) in each scenario				
	A	B	C	D	E
1	68.0	63.8	62.8	56.1	41.3
2	83.0	78.6	76.0	69.8	54.4
3	83.0	76.0	68.5	61.0	39.3
4	82.1	73.3	63.2	54.3	27.8
5	84.6	76.5	66.8	58.9	34.8
6	83.6	75.5	66.8	57.7	31.5

### (c) Simulated scenarios

#### (i) Effects of household structure and contact tracing strategies and parameters on growth rates

To examine the effects of households on transmission and tracing dynamics, we first study the relationship between growth rates and global contact reductions, comparing models with and without household structure. We implement models with no household structure by giving each household in the model a size of one. For models with households, we compare the effectiveness of individual-level tracing with household-level tracing, as described previously.

We estimate the epidemic growth rate by simulating 5000 infected cases to start on day 0 with infectious age 0, which ensures there are enough infections to remove the probability that the epidemic goes extinct immediately and for a sufficiently narrow variability in the growth rate. The epidemic is simulated for 25 days and log-linear regression is used to estimate the growth rate during days 10–25. The first 10 days are discarded as model burn-in, so that visual inspection (electronic supplementary material, figure S1) suggests enough cases of all infectious ages and enough within-household epidemics of various duration have appeared, and for the contact tracing to have been initiated. We first compare the growth rate from a baseline scenario where symptomatic cases initiate self-isolation by themselves and their household members quarantine as described above, but there is no contact tracing initiated upon self-identification of symptoms.

Using a model with household structure and household-level tracing, we examine manual tracing, the effects of a hypothetical tracing app, and two-step tracing across simulations with some parameters set as described in table 1 and other parameters varied as described in table 2. We explore the effects of some households not taking up quarantine or reducing their adherence over time, and we consider initiating tracing on test results rather than on symptoms. The resulting variability observed in estimates of the growth rate is due to the stochastic nature of the simulations and the variability in the parameters, which are sampled from prior distributions as described in table 2.

#### (ii) Simulation of extinction times and probabilities with contact tracing

Using the household-level contact tracing model, we explore the probability that simulated epidemics beginning with a small number of initial infections go extinct over a 2-year period (assuming no additional importations), and the time that it takes them to do so. Because the level of physical distancing has a strong effect on the course of the epidemic, we show the relationship between epidemic end states and extinction times for 0–90% reductions in global contacts with one starting infection and with 100 starting infections. If a branching process model of an epidemic experiences sufficient growth, it will never go extinct since we assume an infinite population and no depletion of susceptibles. As a result, simulated epidemics are stopped once they hit 5000 active infections, as the probability of extinction is effectively zero at this point and exponential growth is achieved. Some parameters are fixed as described in table 1 and others were varied as described in table 2. Additionally, we evaluate the extinction times for scenarios A and E of physical distancing as described in tables 4 and 5.

#### (iii) Backwards contact tracing, with recall decay and digital contact tracing

The NHS England Test and Trace system uses individual-level tracing with tracing initiated on test results [55]. To assess the potential for improving the effectiveness of contact tracing using backwards tracing, we explore the relationship between the growth rate and the number of days prior to symptom onset over which contacts are traced using the individual-level tracing model, i.e. the model where the household structure impacts the infection dynamics, but only the contacts of individuals who test positive are traced and the household members of traced global contact do not quarantine.

We consider individual-level contact tracing with and without a tracing app, and also consider a decay in contact recall as the number of days back to trace increases (10% daily decrease in probability of successfully recalling a contact). If an individual is identified as infectious, then their household is quarantined. Testing is ordered for the individual, and anyone else in the household who has had symptom onset. Upon an individual receiving a positive test result, contact tracing is initiated for contacts that occurred up to X days prior to symptom onset, and up to 7 days post symptom onset, where X is the number of days prior to symptom onset over which contacts are traced, a parameter to be varied. We assumed an unvarying set of parameter values (as shown in table 2) to explore the relationship between growth rates and the number of days back over which to trace.

### (d) Concurrent global contact reductions

While useful to demonstrate the effect of contact tracing in combination with physical distancing, uniform reductions in global social contacts is not inherently an interpretable scenario; the assumption that all households will perform physical distancing equally is expected to be violated in practice. As such, we consider scenarios of different assumed lockdown relaxations by conditioning the reduction in global contacts upon household size, relative to the mean number of global contacts by household size that was estimated in the POLYMOD survey [5]. The lockdown scenario was parametrized using population contact survey data (CoMix) from the lockdown period from end March to end April 2020 [1].

The UK government's plan to relax lockdown consisted of three stages [56], starting with an increase in exercise allowed per day, followed by a phased return of children to schools and opening non-essential retail, followed by places of worship, leisure facilities and hospitality. We loosely based our assumed relaxation scenarios around this plan (tables 4 and 5), though do not model household composition explicitly (table 6).

**Table 6.** Regression coefficients for the effect of contact reductions and contact tracing parameters on growth rates across models with and without household structure and with individual- and household-level contact tracing. We performed 100 simulations for each model, with 5000 starting infections and estimated the growth rates using days 10–25 of the simulation. There is no interpretation of the intercept because we do not simulate scenarios where there is no contact tracing.

parameter	without household structure, test before trace	with household structure, test before trace, household-level tracing	with household structure, test before trace, individual-level tracing
intercept (instantaneous growth rate)	0.240 (0.220, 0.260)	0.240 (0.224, 0.256)	0.235 (0.220, 0.251)
reduction in global contacts (per 10% reduction in global contacts)	−0.0212 (−0.0310, −0.0114)	−0.0132 (−0.0211, −5.35 × 10^−3^)	−0.0151 (−0.0238, −0.0635)
(reduction in global contacts)^2^ (per 10% reduction in global contacts)	−4.02 × 10^−3^ (−8.47 × 10^−4^, 4.37 × 10^−4^)	−5.45 × 10^−3^ (−9.17 × 10^−3^, −1.72 × 10^−3^)	−3.56 × 10^−3^ (−7.32 × 10^−5^, 1.90 × 10^−4^)
(reduction in global contacts)^3^ (per 10% reduction in global contacts)	7.56 × 10^−4^ (1.15 × 10^−5^, 1.50 × 10^−3^)	8.36 × 10^−4^ (2.02 × 10^−4^, 1.47 × 10^−3^)	4.57 × 10^−4^ (−1.53 × 10^−4^, 1.06 × 10^−3^)
(reduction in global contacts)^4^ (per 10% reduction in global contacts)	−7.41 × 10^−5^ (−1.15 × 10^−4^, −3.32 × 10^−5^)	−5.98 × 10^−5^ (−9.50 × 10^−5^, −2.45 × 10^−5^)	−3.87 × 10^−5^ (−7.15 × 10^−5^, −5.78 × 10^−6^)
(probability of having the tracing app)^2^ (per 0.1 increase in probability)	−4.08 × 10^−4^ (−5.66 × 10^−4^, −2.49 × 10^−4^)	−1.23 × 10^−4^ (−2.70 × 10^−4^, 2.36 × 10^−5^)	1.72 × 10^−5^ (−1.18 × 10^−4^, 1.52 × 10^−4^)
probability that a contact made is successfully traced (per 0.1 increase in probability)	−5.43 × 10^−3^ (−7.27 × 10^−3^, −3.59 × 10^−3^)	−5.35 × 10^−3^ (−7.00 × 10^−3^, −3.70 × 10^−3^)	−2.43 × 10^−3^ (−3.82 × 10^−3^, −1.05 × 10^−3^)
mean contact tracing delay (per day)	0.0101 (5.67 × 10^−3^, 0.0145)	5.49 × 10^−3^ (1.38 × 10^−3^, 9.59 × 10^−3^)	1.40 × 10^−3^ (−1.97 × 10^−3^, 4.77 × 10^−3^)
mean testing delay (per day)	9.42 × 10^−3^ (5.01 × 10^−3^, 0.013)	9.71 × 10^−3^ (6.10 × 10^−3^, 0.0133)	5.98 × 10^−3^ (2.38 × 10^−3^, 9.58 × 10^−3^)
untraced case identification probability (per 0.1 increase in probability)	−7.79 × 10^−3^ (−8.72 × 10^−3^, −6.87 × 10^−3^)	−0.0110 (−0.0118, −0.0103)	−9.09 × 10^−3^ (−9.84 × 10^−3^, −8.33 × 10^−3^)

We ran 100 simulations for each relaxation scenario, varying all strategies and parameters across their priors. Adherence and uptake of quarantine is assumed to be perfect, and testing is not required for contact tracing to be initiated. The resulting distribution of the growth rates can be seen in figure 6 and the end states of the simulations are described in table 8.

## 5. Results

### (a) Effects of household structure and tracing on growth rates

**Figure 4.**
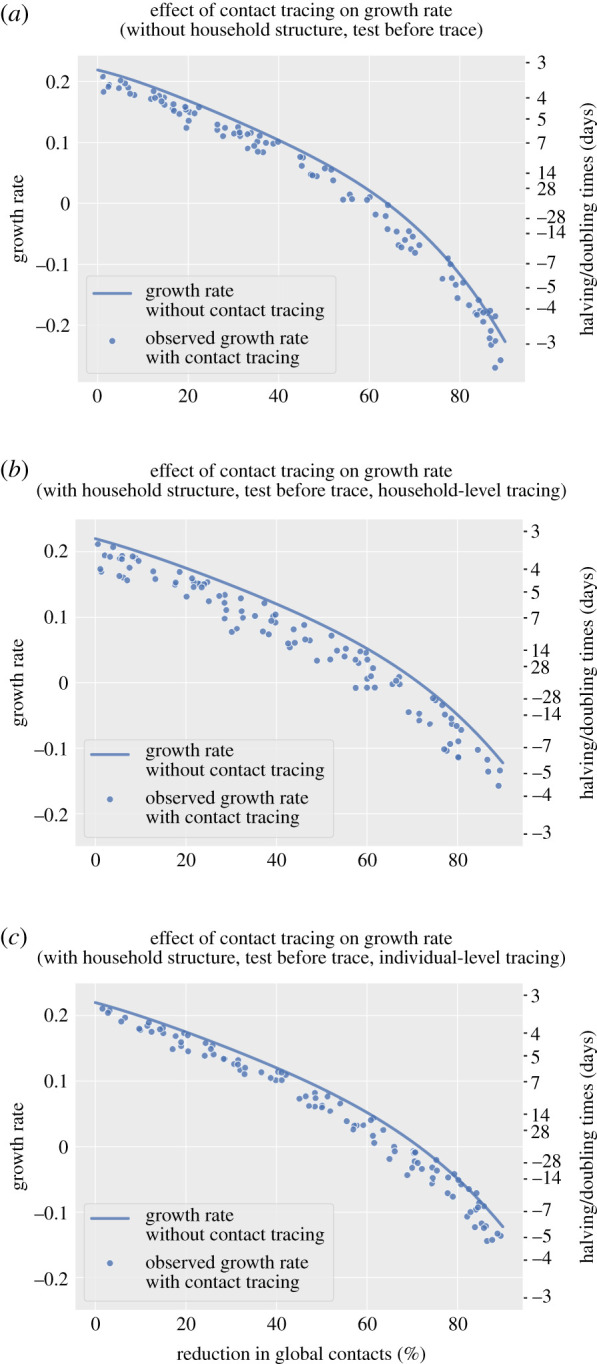
The effect of contact tracing on growth rates of simulated epidemics with and without household structure and by individual-level or household-level tracing strategy. (*a*) No household structure. (*b*) With household structure, household-level tracing. (c) With household structure, individual-based tracing. All scenario required a positive test result to initiate tracing. Negative values on the doubling time axis imply a halving time and a declining epidemic for these values. The growth rate without contact tracing was derived by simulation of the branching process without contact tracing. (Online version in colour.)

In models with household structure (figure 4*b*,*c*), the decline in growth rates associated with higher global contact reductions was less steep compared to epidemics with no household structure (figure 4*a*). A reduction in global contacts is a smaller proportion of total contacts when household structure is modelled explicitly, because there are no local contacts when there is no household structure. To achieve a growth rate of zero, a higher percentage global contact reduction is required in models that include household structure (approx. 70%, no tracing) compared to those that do not (approx. 65%, no tracing).

There was a greater variability in the outcomes of epidemics from models with household structure (figure 4*b,c*) compared to the model with no household structure (figure 4*a*), but the potential gains in controlling the epidemics were greater when household-level tracing was used, for the same range of parameter values (figure 4*b*). The effect of tracing on growth rates was more sensitive to the untraced case self-identification probability in the model with household structure and tracing (0.0110 compared to −7.79 × 10^−3^ for a 0.1 increase in the untraced case self-identification probability). However, when considering a model with household structure but individual-level tracing (figure 4*c*), the effects of contact tracing on the growth rate appeared lower (an approximate 0.02 reduction in the growth rate per day compared to epidemics without tracing).

### (b) Growth rates for a range of household-level contact tracing strategies

**Figure 5.**
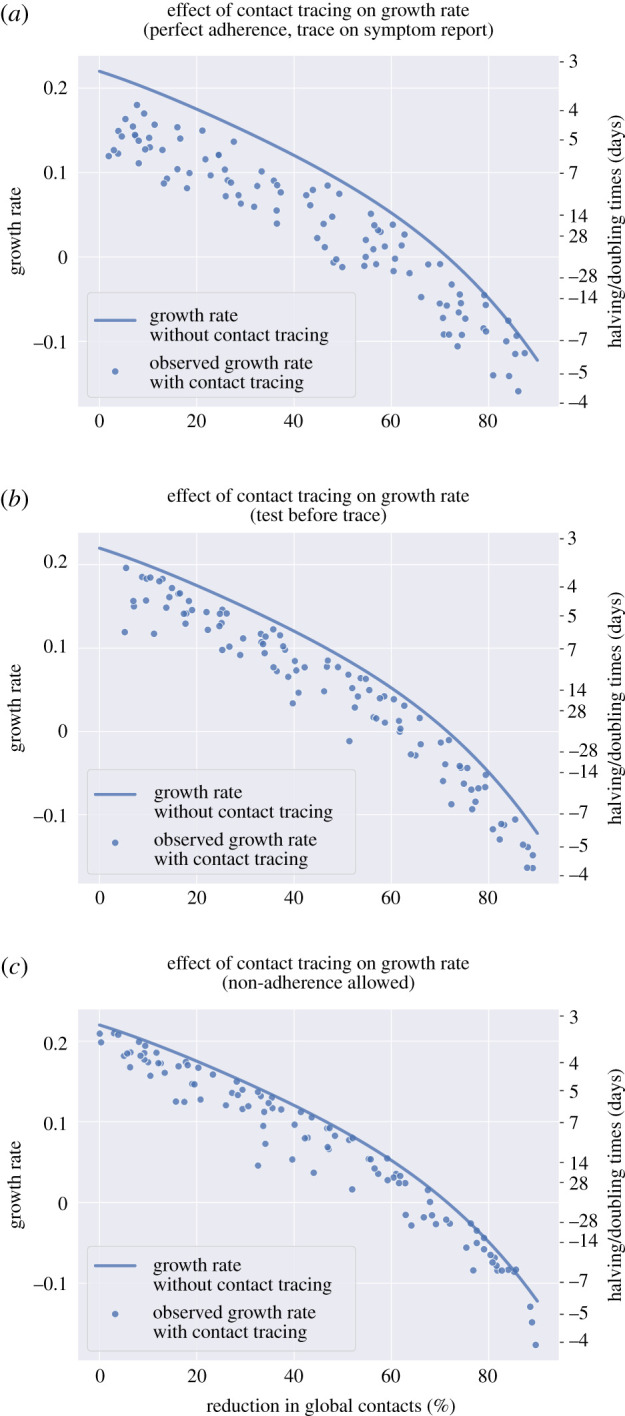
The effect of household-level contact tracing on growth rates of simulated epidemics: tracing initiation and adherence. (a) Tracing initiated without waiting for a test result (initiated on symptom report for untraced cases, symptom onset for traced cases). (b) Positive test result required to initiate tracing. (c) Tracing initiated without waiting for a test result (initiated on symptom report for untraced cases), imperfect adherence to quarantine. For all simulations, household-level contact tracing was used. Two-step tracing was performed at the household level for 50% of simulations. Negative values on the doubling time axis imply a halving time and a declining epidemic for these values. The growth rate without contact tracing was derived by simulation of the branching process without contact tracing. (Online version in colour.)

In a baseline case where individuals with symptoms and their household members quarantine as per UK policy, but there is no contact tracing, the unconstrained growth rate drops below 0 when there is approximately at least a 70% reduction in global contact rates (figure 5). Household-level contact tracing initiated on symptoms report, varying parameter values as described in table 2 (including two-step tracing, unlike in the household comparison simulations above), reduced the growth rate of simulated epidemics by approximately 0.05 d^−1^ compared to baseline across the range of global contact reductions. This controlled the epidemic at global contact reductions of less than 50% for some, but not all, simulated epidemics.

For each 1 day increase in mean testing delay, the growth rate was associated with an increase of 0.0138 (95% CI 0.009–0.018, table 7) when the mean testing delay was varied across the range of 1.5–2.5. This represents a substantial decrease in contact tracing efficacy as testing delays increase.

**Table 7.** Regression coefficients for the effect of contact reductions and contact tracing parameters and strategies on growth rates across models with household structure, household-level tracing and different tracing strategies. Some parameters were fixed as described in table 1 and other parameters were varied as described in table 2. We performed 100 simulations for each model, with 5000 starting infections and estimated the growth rates using days 10–25 of the simulation. Note that the intercept has no interpretation because we do not simulate scenarios with no contact tracing here.

parameter	initiating tracing on symptoms report	initiating tracing on test result	initiating tracing on symptoms report, imperfect adherence
intercept (instantaneous growth rate)	0.289 (0.270, 0.308)	0.220 (0.193, 0.247)	0.3311 (0.310, 0.352)
two-step tracing at the household level (if implemented, compared to not implemented)	−6.70 × 10^−3^ (−9.25 × 10^−3^, −4.16 × 10^−3^)	−0.0106 (−0.0134, −7.78 × 10^−3^)	−6.93 × 10^−4^ (−3.78 × 10^−3^, 2.39 × 10^−3^)
reduction in global contacts (per 10% reduction in contacts)	−0.0214 (−0.0324, −0.0104)	1.95 × 10^−3^ (−0.0146, 0.0185)	−0.0227 (−0.0344, −0.0109)
(reduction in global contacts)^2^ (per 10% reduction in contacts)	−2.33 × 10^−3^ (−7.29 × 10^−3^, 2.63 × 10^−3^)	−0.0103 (−0.0170, −3.62 × 10^−3^)	−2.33 × 10^−3^ (−7.59 × 10^−3^, 2.94 × 10^−3^)
(reduction in global contacts)^3^ (per 10% reduction in contacts)	4.42 × 10^−4^ (−3.90 × 10^−4^, 1.27 × 10^−3^)	1.51 × 10^−3^ (4.73 × 10^−4^, 2.55 × 10^−3^)	5.29 × 10^−4^ (−3.46 × 10^−4^, 1.40 × 10^−3^)
(reduction in global contacts)^4^ (per 10% reduction in contacts)	−4.08 × 10^−5^ (−8.72 × 10^−5^, 5.63 × 10^−6^)	−9.36 × 10^−5^ (−1.48 × 10^−4^, −3.91 × 10^−5^)	−5.11 × 10^−5^ (−9.92 × 10^−5^, −2.91 × 10^−6^)
(probability of having the tracing app)^2^ (per 0.1 increase in probability)	−3.63 × 10^−4^ (−5.30 × 10^−4^, −1.96 × 10^−4^)	−2.78 × 10^−4^ (−4.70 × 10^−4^, −8.79 × 10^−5^)	−1.26 × 10^−4^ (−3.55 × 10^−4^, 1.02 × 10^−4^)
probability that a contact made is successfully traced (per 0.1 increase in probability)	−0.0106 (−0.0122, −8.98 × 10^−3^)	−5.05 × 10^−3^ (−0.705 × 10^−3^, −3.05 × 10^−3^)	−5.92 × 10^−3^ (−8.09 × 10^−4^, −3.75 × 10^−4^)
mean contact tracing delay (per day)	0.0152 (0.011, 0.019)	0.0104 (5.46 × 10^−3^, 1.53 × 10^−2^)	6.66 × 10^−3^ (1.53 × 10^−3^, 0.0118)
untraced case self-identification probability (per 0.1 increase in probability	−0.0227 (−0.0275, −0.0178)	−0.0205 (−0.0255, −0.0155)	−7.29 × 10^−3^ (−0.0130, −1.56 × 10^−3^)
(untraced case self-identification probability)^2^ (per 0.1 increase in probability)	9.35 × 10^−4^ (1.63 × 10^−4^, 1.71 × 10^−3^)	1.30 × 10^−3^ (4.81 × 10^−4^, 2.12 × 10^−3^)	1.04 × 10^−4^ (−8.49 × 10^−4^, 1.06 × 10^−3^)
mean testing delay (per day)	n.a.	0.0138 (9.17 × 10^−3^, 0.0184)	n.a.
probability a household will take up isolation (per 0.1 increase in probability)	n.a.	n.a.	−9.76 × 10^−3^ (−0.0109, −8.61 × 10^−3^)

After global contact reductions, the parameter with the greatest effect on epidemic growth across scenarios was the probability that infections are identified in the absence of contact tracing (table 7). This is perhaps unsurprising since all other tracing parameters depend on this.

Two-step tracing at the level of households had a greater effect when tracing was initiated on a positive test result rather than on symptoms (table 7). Fundamentally, two-step tracing is a strategy that improves the speed of the contact tracing process by tracing contacts of contacts of an infected case, as opposed to waiting until a one-step generation contact develops symptoms and tests positive. As such, the improvement from two-step tracing is greater when it is used to offset the slowdown caused by testing every individual before propagating contact tracing. We assume that it does not affect the probability that a contact is successfully traced, though if a model of recall is considered there can be interactions with the probability a contact is recalled, since first generation contacts would be asked to recall their contacts earlier. No appreciable effect of two-step tracing was observed in the model including non-uptake and waning adherence to isolation and quarantine: its slight gains appear to have been eroded by the non-adherence effects. In particular, adverse effects might occur in the model when adherence to quarantine decreases over time: the two-step contact tracing strategy would result in individuals being quarantined earlier, so when individuals start non-adhering to quarantine, they may have just reached the most infectious part of their infectious period, with an overall potentially detrimental effect of the strategy. In general, though, non-uptake of isolation or quarantine was associated with higher growth rates; however, a waning effect on adherence to isolation or quarantining (i.e. leaving early) was not.

The tracing delay was less important when considering tracing on test results rather than on symptoms and when considering non-uptake and non-adherence to quarantine (table 7). The tracing delay occurs relatively late in the transmission process, after the time until case identification, the fact that not all cases are identifiable (e.g. asymptomatic) and the testing delay, so the relative gains or losses that can be seen at this stage in the process are more limited**.**

### (c) Backwards contact tracing and recall

We consider the role that implementing backwards tracing might play in improving the effectiveness of England's Test and Trace policy, by varying the number of days prior to symptom onset over which to trace contacts.

**Figure 6.**
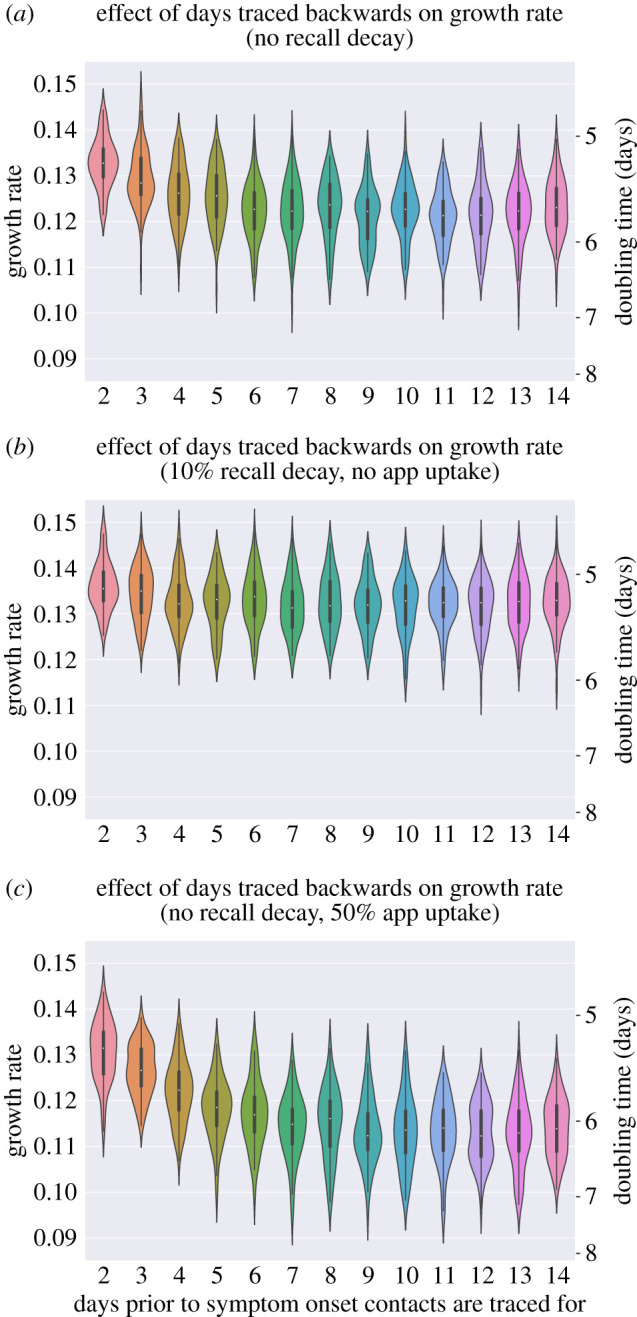
Backwards tracing: effects on growth rates of increasing the days prior to symptom onset over which tracing is performed. (*a*) No recall decay and no digital contact tracing app. (*b*) Recall probability decaying at 10% each day and no digital contact tracing app. (*c*) No recall probability decay and 50% uptake of the digital contact tracing app. (Online version in colour.)

We find that increasing the number of days prior to symptom onset over which contact tracing is performed improves the efficacy in reducing epidemic growth rate (figure 6*a*), especially when app uptake is high (50%, figure 6*c*) resulting in more digital contact tracing which has no contact tracing delay and more contacts successfully traced. There is a linear decrease in the growth rate up until around 8–10 days, after which no more gains appear achievable.

However, identified cases might struggle to remember contacts they have had back in the past. We find that including a daily 10% reduction in the probability of recalling a contact in the model erodes the gains of backwards contact tracing almost completely, such that there is little difference in the growth rate of the epidemic according to the number of days pre-symptom onset a case's contacts are traced (figure 6*b*). For the index case in a contact tracing chain to have occurred, the index case must have had symptom onset, followed by a symptom reporting delay and a possible testing delay before the backwards tracing attempt to the infector is initiated, a combination that would have already led to significant decay in the ability to recall. A further contact tracing delay would ensue until the infector is reached, who would then have to recall the other contacts that they made at around the time they contacted the index case. As a result, the recall decay significantly impacts the probability that backwards and then forward tracing is successful. In general, however, the overall reduction on the growth rate of the epidemic appears limited.

### (d) Lockdown exit scenarios

**Figure 7.**
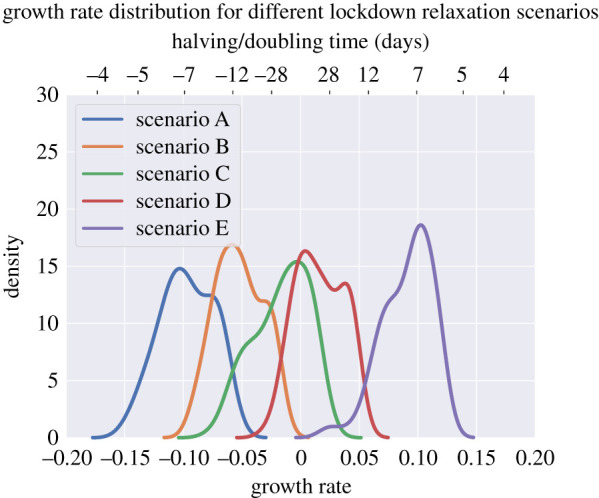
Distributions of epidemic growth rates under the different lockdown relaxation scenarios. Scenarios are as described in tables 4 and 5, with scenario A on one extreme representing a small increase in school and workplace contacts, and scenario E on the other extreme representing a larger increase in work contacts, as well as resumption of school contacts and resumption of most leisure contacts. Negative values on the doubling time axis imply a halving time. (Online version in colour.)

In figure 7, we plot the observed distribution of the growth rates under the assumed scenarios described in tables 4 and 5. With household-level contact tracing and global contact patterns analogous to scenarios A and B, i.e. with small increases in school and workplace contacts for both and an additional 10% increase in leisure contacts for B, the growth rate for all simulations remained below zero. However, for scenarios C and D, the results for positive or negative growth were mixed, with nearly all simulated epidemics with contact levels analogous to scenario E finding positive growth of the epidemic.

### (e) Extinction times

We simulated epidemics for a duration of 2 years (730 days) to estimate the proportion of epidemics that go extinct, and the time taken to do so, varying contact tracing parameters as in table 2.

**Figure 8.**
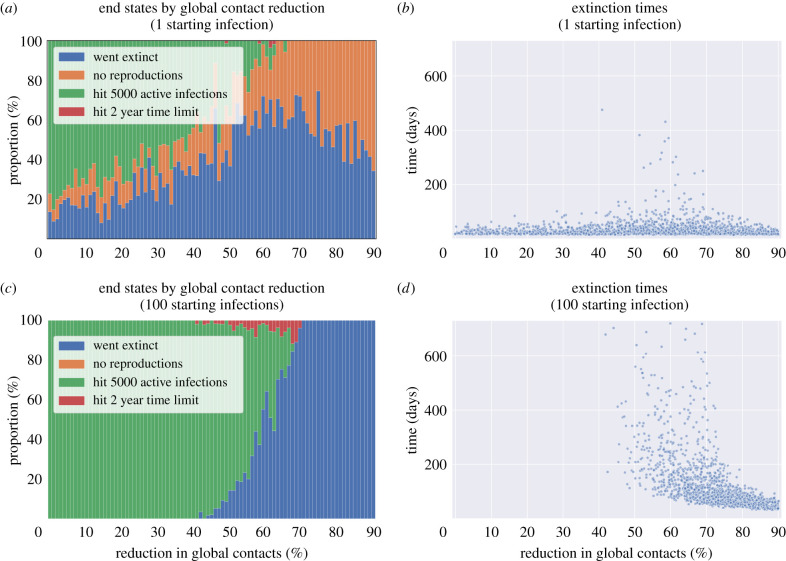
End states and extinction times of simulated epidemics for reductions in global contacts compared to pre-pandemic levels and number of cases in the first generation of the epidemic. (*a*) End states for a single initial case. (*b*) Extinction times for a single initial case. (*c*) End states for 100 initial cases. (*d*) Extinction times for 100 initial cases. (Online version in colour.)

We define several possible end states for an epidemic; ‘Never grew’, where the first generation produces no offspring; ‘Grew exponentially’, where we stop the simulation once 5000 active infections is reached, as at this point we assume the probability of extinction is zero due to the size of the epidemic; ‘Timed out’, where we stop simulating epidemics that were not extinct after 2 years nor hit 5000 active infections; and finally ‘Went extinct’, where the first generation produces some offspring, but the number of active infections then drops to zero and no more infection events occur. Which end state occurs depends strongly on the global contact reductions (figure 8). For a single infection, approximately half of epidemics go extinct if the global contact reduction is at least 50%. For 100 starting infections approximately half of simulated epidemics go extinct at a contact reduction of approximately 65%, with variation in the end states occurring between 50 and 70%. That is, below a 50% global contact reduction, contact tracing and quarantine does not bring the epidemic under control, in line with what was already discussed in terms of growth rates (figure 5*a*). Above the 50–70% range, the mean and variance in extinction times reduces as all epidemics rapidly go extinct.

**Figure 9.**
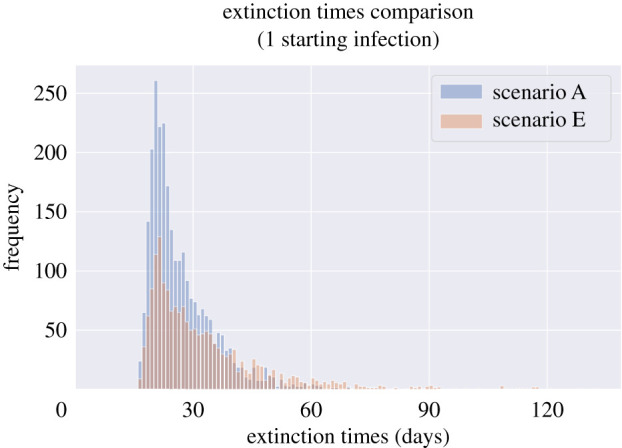
Extinction times of simulated epidemics started with a single infection that reproduced at least once for the two extreme scenarios of social distancing relaxation described in tables 4 and 5. Scenario A represents a small increase in school and workplace contacts, and scenario E represents a larger increase in work contacts, as well as resumption of school contacts and resumption of most leisure contacts. All other parameters vary as described in table 2. Extinction times can be significant, even when there is a high level of physical distancing and only a single starting infection. (Online version in colour.)

Considering lockdown exit scenarios linked to increases in work, school and leisure contacts, from scenario A to E described in tables 4 and 5, we found that the likelihood of an initial infection causing secondary infections increased (table 8), and that once this occurred, there were more epidemics that could not be controlled. Among epidemics that were controlled, there were more simulated epidemics which took longer to go extinct in scenario E compared to A (figure 9), though even in scenario A interventions clearly need to be kept in place for a long time (even ignoring the chances of external introduction).

**Table 8.** End states of simulated epidemics with a single initial case for assumed scenarios of physical distancing relaxation. Scenarios are as described in tables 4 and 5, with scenario A on one extreme representing a small increase in school and workplace contacts, and scenario E on the other representing a larger increase in work contacts, as well as resumption of school contacts and resumption of most leisure contacts.

scenario	% epidemics that did not reproduce	% epidemics that went extinct	% epidemics that grew exponentially	% epidemics that timed out
A	45.8	54.2	0	0
B	39.8	60.2	0	0
C	35.3	64.0	0.3	0.4
D	30.4	61.6	7.8	0.2
E	20.6	36.1	43.3	0

When we considered epidemics with no physical distancing, the probability of epidemic extinction arising from a single initial infected individual was much lower, even given contact tracing with 100% adherence to quarantine (not shown here).

## 6. Discussion

We find that implementing a contact tracing, isolation and quarantine policy could contribute to controlling the SARS-CoV-2 epidemic if lockdown levels of physical distancing are partially relaxed, but not if they are relaxed completely. In our household-structured branching process model, none of our strategies to improve contact tracing would prevent the re-emergence of epidemic growth once global contacts relative to pre-pandemic times are reduced by less than approximately 40%. In our simulations, we find that the probability that an untraced case is identified is one of the most important parameters for contact tracing and quarantine effectiveness. We have explored strategies that could be used to make contact tracing more effective. Notably, household-level tracing was more effective in reducing the growth rate of epidemics than individual-level tracing, though this and other strategies need to be considered in terms of the increased number of individuals who would need to be traced and go into isolation. The extinction times of some simulated epidemics, even with very small starting numbers of infections, could be potentially very long, which underlines the need to account for sustainability of interventions over long periods of time, particularly if local SARS-CoV-2 elimination is deemed a goal. Consideration should be given to our findings that effectiveness gains could be eroded when the population is effectively unable or unwilling to take up isolation and quarantine, and by implementation challenges, such as contact recall difficulties in the case of backwards tracing.

Countries that have suppressed the number of SARS-CoV-2 cases have done so with a combination of policies, including different types of contact tracing and varied levels of suggested and enforced physical distancing policies. Our findings that contact tracing, isolation and quarantine are unlikely to be effective on their own in suppressing the SARS-CoV-2 epidemic are consistent with those of other models [23,28,57]. The contribution of contact tracing and isolation in our model was most significant when global contacts were reduced to 40–70% of their pre-pandemic levels, above which growth was not suppressed and below which very few epidemics took off (contingent on the transmission parameters we assumed). Further evidence on the transmissibility among different groups, types of contacts and in different settings will allow a more nuanced interpretation of which physical distancing policies are most important to retain.

After the global contact rate, the probability that a case is identified was the next most important model parameter to the effectiveness of contact tracing, isolation and quarantine. In our model, this parameter reflects a combination of biological factors (e.g. proportion of asymptomatic and subclinical infections), testing policies (who is tested and when in their infection), and test characteristics (sensitivity and specificity). Estimating the proportion of cases who are identified overall is challenging; studies in other European countries indicate that the overall proportion of cases who are identified was low at time of writing (20% in Spain, 10% in France after their first lockdown of 2020 [58,59], though widespread testing in the second half of 2020 has possibly increased these proportions). Our findings suggest that even with a contact tracing, isolation and quarantine system in place, additional case identification methods would be beneficial, for instance frequent screening of high-risk groups. This is analogous to the approaches taken for the control of sexually transmitted infections, which uses both screening of high-transmission risk groups and tracing (partner notification), but requires an understanding of the links between ‘high’ and ‘low’ risk individuals and how their definition changes over the course of the epidemic [60].

Once the number of global contacts and the proportion of cases discovered are in a range in which contact tracing, isolation and quarantine could hypothetically control the epidemic, the speed of the process relative to the speed of transmission—fast in the case of SARS-CoV-2—is important. Strategies to either minimize or eliminate delays, including symptom-initiated tracing rather than waiting for test results and digital contact tracing were more effective in suppressing epidemic growth compared to manual tracing (at least under the assumption of high app uptake, in line with what others have found [53]). Other strategies to ‘get ahead’ of transmission, including earlier isolation of contacts via two-step contact tracing and isolation or concurrently isolating a contact's whole household, also reduced growth rates but less significantly so. Backwards tracing improved effectiveness up to tracing 8–10 days prior to symptom onset assuming no change in the probability of successfully tracing contacts, after which presumably the time delay incurred before tracing ‘forwards’ again on previously missed transmission branches becomes too long to ‘catch-up’.

However, for each of the strategies that could theoretically improve the effectiveness of contact tracing, there are implementation challenges that could erode their effectiveness. When considering a possible decrease in successfully tracing contacts back in time to enable backwards tracing, we found that a plausible ‘worst case’ assumption in declining recall of contacts could almost cancel out any gains made. Many outbreaks occur in settings with specific environmental conditions, so implementing backwards tracing by asking individuals where they have been, instead of who they met, could help facilitate the discovery of clusters of infections.

If uptake of isolation policies is less than 100% and if adherence to isolation wanes over the period of isolation, during which time household transmission could continue, we found that effectiveness in suppressing epidemic growth degraded. Monitoring of adherence and policies to enable and support people to take up and adhere to testing, tracing and isolation are crucial.

Finally, in our analysis, the time it would theoretically take to eliminate SARS-CoV-2 could be in the order of months or years given slight relaxations of lockdown. These long theoretical extinction time estimates assume no further importation of cases into the population, which is not likely in practice, suggesting that control policies would be required over a long timeframe.

### (a) Strengths and limitations

One of the key elements that set our model apart from most other published studies of contact tracing is the explicit presence of the household structure, which enables more realistic transmission dynamics and explicit modelling of household quarantines and isolation and the strategic options these create. Interactions between physical distancing and household structure are important to consider—a 90% reduction in global contacts does not necessarily correspond to a 90% reduction in transmission, as within-household epidemics will continue to spread. However, we have not explicitly modelled increased levels of local transmission following high levels of global contact reductions causing individuals to spend more time at home, because an increase in the number of local contacts was not observed in a population survey after the first lockdown [1]. We found that models that do not include the household structure explicitly risk underestimating the potential impact of an individual-level tracing strategy, nor are they able to model household-level contact tracing strategies, which prove to be more effective when used in a real population with households. However, we do not include other important clustering in our model that would connect households, such as workplaces or schools, which are important to consider as children return to school and more workers are encouraged to stop working from home.

The branching process structure of our model means that we cannot account for global susceptible depletion, though local within-household susceptible depletion is accounted for. For example, most individuals likely repeat the same global contacts each day resulting in a finite pool of individuals they could infect, such as office workers who repeat contacts with their colleagues each day [61]. If the pool of individuals a case could infect is very small, which we might expect during lockdown, then it is possible the model is overestimating the number of secondary infections from a case since it does not allow for the depletion of the pool of susceptibles contacted outside the household. This would result in the model overestimating the growth rate at high levels of physical distancing, and possibly underestimating the extinction probability. At low levels of physical distancing, where outside-household contact pools are presumably larger, this is less likely to be a concern.

As the pandemic continues, modelling a level of immunity in the population will be required. In the UK, the ONS has estimated seroprevalence from antibody testing on blood donors at approximately 6% in England (as of 23 August 2020), with regional variations [19]. It is also not yet clear how durable the immune response to a repeat exposure is, and how this might vary by severity of the initial infection and other factors. Levels of immunity will continue to be heterogenous by region and sociodemographic factors, which need to be accounted for. A relatively simple model extension could be employed to account for infection-acquired immunity: as we expect larger households to be depleted fastest, given the more members of a household there are, the greater the rate of importing the infection into that household, one could simply approximate the exact epidemic process by shifting the size distribution of newly infected households towards lower values, based on total numbers of households of each size in the simulated regions and counting for those household that have already been infected. This may result in strategies that are designed to take advantage of the influence of larger households on both transmission and contact tracing, such as household-level contact tracing, decrease in effectiveness over time as the infection gradually shifts from larger to smaller households. However, an explicit network structure or an agent-based model would be needed to correctly account for multiple introductions on SARS-CoV-2 in the same households.

When considering high levels of immunity, contact network effects would further come into play at the individual level, as elements of the population who make a large number of contacts on a regular basis will have an increased likelihood of immunity. Our model is currently limited to households, but other settings such as schools and workplaces should be considered. We have not explicitly modelled ‘support bubbles’, whereby households of single individuals have been allowed to function as one larger household under physical distancing restrictions. Individual-level heterogeneities, such as age, specific vulnerabilities to severe infection or characteristics associated with increased exposure and transmission, and the relationship to household structure were not included in the model. An underlying assumption of our model is homogenous mixing between households, as such network effects are not included in this model, meaning we could be underestimating the effectiveness of contact tracing, which preferentially removes individuals with many contacts (those with ‘high degree’) [11], though this might be subject to other epidemic characteristics and contact tracing performance [36,53,62].

Relatedly, we do not explicitly model the ‘costs’ to different contact tracing, isolation and quarantine strategies and choices, including the number of people required to isolate per identified case. This will vary according to contact patterns in the population at a given point in time, and the impact on individuals and society, for instance, via the isolation of key workers, will also vary. The risk of digital tracing of identifying a large number of non-epidemiologically relevant contacts is another potential problem to consider. Previous studies of quarantine orders vary widely in their findings as to adherence patterns over time [25]. We also do not model indirect benefits to reducing transmission that contact tracing could bring, such as improved surveillance and understanding of transmission patterns that could enable better timed and targeted interventions.

We conclude that while there are strategies to improve the effectiveness of contact tracing, isolation and quarantine on epidemic growth, contact tracing as modelled here will not be effective in suppressing epidemic growth on its own, given current understanding about transmissibility of the virus, without continued reductions in out-of-household contacts. To be effective, contact tracing, isolation and quarantine need to be performed with minimal delays and a high degree of accuracy. Further, it is important to consider support to uptake and adherence to policies, and to better understand potential trade-offs between strategies that reduce epidemic growth but which might have a negative effect upon adherence, such as digital tracing. Thought needs to be paid to practical implementations in order to gain the theoretical effect. It is likely that, as more is understood about transmission settings, dynamics and testing, contact tracing, isolation and quarantine can continue to be refined and will remain an important part of a targeted approach to the control of SARS-CoV-2, as well as of future infections.

## 7. In context

This model and further development of it have been used to inform the design and expectations of the effectiveness of Test, Trace and Isolate (TTI) policies. The authors have been reporting on this topic to the Scientific Pandemic Influenza Group on Modelling (SPI-M) and the Scientific Advisory Group for Emergencies (SAGE) from the beginning of May 2020 until the present time (March 2021), and will continue throughout the rest of the pandemic. During the first UK national lockdown (March–May 2020) falling SARS-CoV-2 death, hospitalisation and case numbers were the result of strict physical distancing policies and, once these policies were eased, the expectation was that prevalence would rise again. There was a need to assess the potential effectiveness of various contact tracing strategies on controlling epidemic growth while physical distancing measures were gradually relaxed, and to assess optimal implementation choices.

Early modelling analyses had found that contact tracing would very likely struggle to control epidemic growth of SARS-CoV-2 on its own and would need to be implemented alongside other physical distancing measures [9,57]. The commonly used ‘forwards’ contact tracing strategy aims to find and quarantine exposed contacts of a confirmed or probable case before they have had an opportunity to transmit. However, for SARS-CoV-2 the significant proportion of pre-symptomatic and asymptomatic transmission makes this very challenging. It was therefore important to assess potential modifications to the process that could improve the effectiveness.

The model we use in the analyses presented here was developed from January 2020 onwards, with a focus upon household structure, and the interaction between household structure and contact tracing. Households play an important role in the transmission of respiratory infections given the close and repeated nature of contacts within them. However, household structure should also increase the efficiency of contact tracing, given that the tracing of household contacts is expected to be easier when compared to out-of-household contacts, resulting in the rapid quarantine of a whole household when a case is discovered. Further, the explicit implementation of household structure allows us to assess the utility of strategies that take advantage of the household structure to benefit the contact tracing process–the ‘household-level’ contact tracing strategy we refer to in the article, which treats household as the units of contact tracing, as opposed to individuals. In analyses sent to SPI-M in early May 2020, we assessed the effects on epidemic growth of relaxing restrictions on out-of-household contacts broadly in alignment with assumed lockdown easing scenarios, and the probabilities and timescales with which household-level contact tracing, in combination with remaining physical distancing policies, would reduce the epidemic to extinction in the absence of additional importations. The latter investigations on epidemic extinction were not a full investigation into the eradication or elimination prospects of SARS-CoV-2, as these depend on other factors such as importation, but were intended to bring broad insight to perceptions of the period of time over which restrictions would need to be in place. At the same time, we explored the possible effects of a range of potential strategies to improve the potential effectiveness of TTI including initiation of tracing on symptoms, ‘two-step’ tracing and quarantining of contacts-of-contacts, and a hypothetical contact tracing app.

At the end of May 2020, the structure and policies of NHS Test and Trace were announced and we modified our model to reflect such information. The approach did not include household-level tracing and required a positive test in order to initiate tracing, rather than tracing on symptoms alone. As modelled, the existing policy should have a reduced effectiveness compared to household-level tracing or symptom-initiated tracing. These strategies could potentially be more effective at reducing epidemic growth, but at the cost of an increase in the number of individuals who have to quarantine [30]–a quantity that our model is less well-suited to measure given the lack of non-infected individuals. Once adapted to the UK policy, we used our model to assess the effectiveness of a ‘backwards tracing’ strategy, which aims to identify a case's infector and allows for the identification of more branches of transmission when compared to forwards tracing. We, and other groups [35,36], found that in theory it could improve contact tracing, but also that implementation challenges could reduce this effect. Ultimately, the NHS Test and Trace programme in England continued to use forwards tracing for standard cases not associated with a particular list of high-risk settings.

The publication in this issue includes analyses up until August 2020. Since then, the model has been used to investigate and report via SPI-M/SAGE a range of other TTI policy choices including: out-of-household isolation of cases or quarantine of vulnerable household members; trade-offs between lengths of quarantine and uptake/adherence to symptomatic testing and quarantine, in recognition of challenges in uptake and adherence to policies as implemented (along with another contact tracing model described here [63]); and the implications for TTI effectiveness of limited testing capacity, an analysis that clarified the need to maintain testing capacity where prevalence is still low to avoid local epidemics spiralling out of hand precisely where contact tracing is more likely to succeed at achieving control. More recent work involves investigations into daily contact testing using lateral flow tests in lieu of quarantine [64], testing and quarantining strategies of travellers returning to households from abroad [65], and potential changes to the symptom criteria required for PCR-testing.

Our investigations have highlighted the importance of interdisciplinary collaboration with behavioural and social scientists, and our work has fed into interdisciplinary reports [66]. As indicated in this paper, the extent to which symptomatic testing is taken up in the presence of symptoms and the extent to which individuals are enabled to adhere to isolation and quarantine measures are critical; subsequent behavioural research [67–69] has been widely varying, but often indicative of low adherence to policies. Throughout the pandemic, it has been clear that support and communication for members of the public to get tested, self-isolate and quarantine is extremely important, as these are the foundations of a successful contact tracing-based public health response.

The underlying mathematical assumptions of the model do not allow for the modelling of immunity in a population, both infection- and vaccine-acquired, and this has lately motivated moving onto other model structures. Overall, the model has proven a useful and flexible tool to provide rapid responses to emerging policy questions, while retaining the important aspects and complexity induced by household structure. We are continuing to develop the code for release as a fully featured Python library, for use in rapid response to future epidemics.

## Data accessibility

The datasets/code generated during and/or analysed during the current study are available in the HouseholdContactTracing repository https://github.com/martyn1fyles/HouseholdContactTracing, with the exception of UK-specific line-list data. These data are provided by Public Health England under a data sharing agreement and we are unable to share these data.
